# Inverse Association of Circulating SIRT1 and Adiposity: A Study on Underweight, Normal Weight, and Obese Patients

**DOI:** 10.3389/fendo.2018.00449

**Published:** 2018-08-07

**Authors:** Stefania Mariani, Maria R. di Giorgio, Paolo Martini, Agnese Persichetti, Giuseppe Barbaro, Sabrina Basciani, Savina Contini, Eleonora Poggiogalle, Antonio Sarnicola, Alfredo Genco, Carla Lubrano, Aldo Rosano, Lorenzo M. Donini, Andrea Lenzi, Lucio Gnessi

**Affiliations:** ^1^Section of Medical Physiopathology, Food Science and Endocrinology, Department of Experimental Medicine, Sapienza University of Rome, Rome, Italy; ^2^Italian Hospital Group, Center for the Treatment of Eating Disorders and Obesity “Villa Pia”, Guidonia, Italy; ^3^Department of Molecular Medicine, Sapienza University of Rome, Rome, Italy; ^4^Department of Surgical Sciences, Policlinico Umberto I, Sapienza University of Rome, Rome, Italy; ^5^Roman Academy of Public Health, Rome, Italy

**Keywords:** sirtuins, circulating SIRT1, adiposity, anorexia, obesity, body fat mass

## Abstract

**Context:** Sirtuins (SIRTs) are NAD+-dependent deacetylases, cellular sensors to detect energy availability, and modulate metabolic processes. SIRT1, the most studied family member, influences a number of tissues including adipose tissue. Expression and activity of SIRT1 reduce with weight gain and increase in conditions of starvation.

**Objective:** To focus on SIRT1 plasma concentrations in different conditions of adiposity and to correlate SIRT1 with fat content and distribution, energy homeostasis and inflammation in under-weight, normal-weight, and obese individuals.

**Materials and Methods:** 21 patients with anorexia nervosa, 26 normal-weight and 75 patients with obesity were evaluated. Body fat composition by dual-energy X-ray absorptiometry, ultrasound liver adiposity, echocardiographic epicardial fat thickness (EFT), inflammatory (ESR, CRP, and fibrinogen), and metabolic (FPG, insulin, LDL- and HDL-cholesterol, triglycerides) parameters, calculated basal metabolic rate (BMR) and plasma SIRT1 (ELISA) were measured.

**Results:** SIRT1 was significantly higher in anorexic patients compared to normal-weight and obese patients (3.27 ± 2.98, 2.27 ± 1.13, and 1.36 ± 1.31 ng/ml, respectively). Linear regression models for each predictor variable adjusted for age and sex showed that SIRT1 concentration was inversely and significantly correlated with EFT, fat mass %, liver fat content, BMR, weight, BMI, WC, LDL-cholesterol, insulin, ESR. Stepwise multiple regression analysis revealed that age and EFT were the best independent correlates of SIRT1 (β = −0.026 ± 0.011, *p* = 0.025, and β = −0.516 ± 0.083, *p* < 0.001, respectively).

**Conclusions:** Plasma SIRT1 shows a continuous pattern that inversely follows the whole spectrum of adiposity. SIRT1 significantly associates with EFT, a strong index of visceral fat phenotype, better than other indexes of adiposity studied here.

## Introduction

Sirtuins (SIRTs) are nutrient sensing, metabolic regulators, and chromatin silencers (1). SIRT1, the most-studied SIRT, is best known for mediating lifespan extension by consistently improving health during aging. Results mainly derived from animal studies show that SIRT1 protects against or delays the onset of metabolic diseases, neurodegeneration, cardiovascular diseases, and some types of cancers (1, 2). SIRT1 modifies the acetylation status of many different targets in cytoplasm, mitochondria, and nucleus, and it carries out its protective roles by activating key transcription factors, improving lipid metabolism, reducing inflammation, and acting as a tumor suppressor by preserving genomic integrity. SIRT1 plays also an essential role in adaptive metabolic and endocrine responses (3). Several metabolic disorders such as liver steatosis, diabetes, and obesity associate with defects in SIRT1 pathways. Obesity is associated with low NAD(+)/SIRT pathway expression in subcutaneous adipose tissue of BMI-discordant monozygotic twins, highlighting a strong relationship of reduced SIRTs expression with inflammation, insulin resistance, and impaired mitochondrial homeostasis (4). Visceral adiposity negatively correlates with SIRT1 expression (5, 6). Accordingly, we have previously shown an inverse association between plasma SIRT1 and ectopic fat distribution in patients affected by obesity (7, 8) in particular with epicardial and liver fat depots, both typical examples of visceral fat with particularly detrimental effect because of localized and systemic toxic effects (9, 10). Although the main source of circulating SIRT1 is not known (11–13), these results indicate that the negative metabolic effects of obesity could be related, at least in part, to the reduced levels of SIRT1 in the blood. Moreover, what regulates circulating SIRT1 vs. tissue SIRT1 is still unknown.

Conversely, SIRTs tissue enzymatic activity increases in conditions of nutrient depletion and starvation. SIRT1 expression rises in cultured cells, and in multiple tissues of mice after overnight or 24 h fasting (14, 15). An increased expression of SIRT1 is seen after long periods of calorie restriction (CR) in mice (16). Analogously, in man, 30 days and 7 weeks of CR cause a rise of tissue and plasma levels of SIRT1, respectively (17, 18). Indeed, SIRT1 has been identified as a novel factor responsible for some beneficial effects of CR, and previous studies showed that weight loss induces an increase in tissue and circulating SIRT1 levels in obese patients (19, 20).

Therefore, SIRT1 may act differently in states of nutritional excess compared with states of nutritional deprivation.

Circulating SIRT1 has not been studied yet in underweight individuals or in subjects who restrict eating. Thus, in relation to its opposite behavior in condition of hyper- or hypo-nutrition, we evaluated SIRT1 blood concentration, body fat composition, markers of energy homeostasis, inflammation and some metabolic parameters in underweight, normal-weight, and obese individuals, i.e., in subjects with defect or excess of body fat mass. The aim of the study was to investigate the plasma SIRT1 concentration across the whole spectrum of adiposity, and its relationship with fat distribution and metabolic, inflammatory and energy settings.

## Subjects and methods

Study participants were recruited among subjects referring to the High Specialization Center for the Care of Obesity (CASCO), Department of Experimental Medicine, “Sapienza” University of Rome, and from the Italian Hospital Group, “Villa Pia,” Guidonia, Italy, from January 2015 to February 2017. The study was approved by the ethical committee of the Sapienza University of Rome, Policlinico Umberto I, and was concordant with Helsinki Declaration. Each patient gave a written informed consent before admission to the study.

Over the 2 year recruitment period, a total of 50 patients with anorexia nervosa (AN), 400 obese individuals and 150 normal-weight consecutive subjects were screened. After screening, 122 patients were included. 21 underweight patients with AN based on the diagnostic criteria of the DSM-5 (3 males, 18 females, age range 16–68 year, BMI range 10.63–20.23 Kg/m^2^); 26 normal-weight control individuals (7 males, 19 females, age range 20–59 year, BMI range 20.22–24.83 kg/m^2^); 75 patients affected by obesity (19 males, 56 females, age range 18–65 year, BMI range 31.36–59.0 kg/m^2^). The subjects were excluded either on the basis of the criteria reported below or declined to participate. A portion of the obese and normal-weight patients were included in two previous studies (7, 8).

The exclusion criteria were: uncontrolled hypertension, heart diseases, lung diseases, type 1 diabetes, uncontrolled type 2 diabetes, corticosteroids for systemic use, any medication potentially affecting body weight or body composition, cirrhosis and other chronic liver diseases, acromegaly, hypothyroidism, acute illness, current or past presence of hepatitis B surface antigen and antibody to hepatitis C virus, excessive alcohol intake (≥140 g/week for men or 70 g/week for women).

All patients underwent complete medical examination and anthropometric measurements [body weight (kg), height (m), waist circumference (WC) at the level of umbilicus (cm)]. Body weight was measured by Tanita BWB-800A digital medical scale (Tanita Corporation, Arlington Heights, IL, USA). BMI was calculated by the formula weight (kg)/height(m)^2^.

Fasting plasma glucose (FPG, mg/dl) and insulin (mU/L), total cholesterol (TC, mg/dl), high-density lipoprotein (HDL)-cholesterol (mg/dl), low-density lipoprotein (LDL)-cholesterol (mg/dl), triglycerides (TG, mg/dl), erythrocyte sedimentation rate (ESR, mm/h), C-reactive protein (CRP, μg/L), fibrinogen (g/L), and SIRT1 (ng/ml) were assessed after a 12-h overnight fast. Plasma samples for SIRT1 analyses were frozen at −80°C until measurement. Because intermittent fasting might influence the circulating levels of SIRT1, a special attention was paid to withdrawing the blood at the same 12 h time interval from the last meal for all patients. Dual energy X-ray absorptiometry (DXA) body composition, echocardiographic epicardial fat thickness measurements (mm) and liver adiposity by ultrasound were also recorded.

The estimated BMR value was calculated using the Harris & Benedict equation and expressed in kcal/day. Following the equations for men and women:

$$\begin{array}{l} \text{Men }=\;66.4+13.75\times \left(\text{Wt}\right)+5\times \left(\text{Ht}\right)-6.8\times \left(\text{Age}\right)\\ \text{Women }=\;655+9.6\times \left(\text{Wt}\right)+1.85\times \left(\text{Ht}\right)-4.7\times \left(\text{Age}\right) \end{array}$$

### SIRT1 assay

SIRT1 was determined by a monoclonal antibody-based ELISA method using a commercially available human SIRT1 ELISA kit (MyBioSource, Cod. GDMBS705558) with an inter- and intra-assay coefficient of variation of 10 and 8%, respectively, and a detection limit of 0.039 ng/mL.

Microtiter plates were coated with equal amount of primary mouse anti-human SIRT1 monoclonal IgG. 100 μL standard and plasma samples were pipetted in each well and the protocol was followed by using secondary avidin conjugated horseradish peroxidase. The formation of horseradish peroxidase was measured at 405 nm using ELISA reader (Quanta Biotech, UK). Seven different concentrations of purified SIRT1 (0.15, 0.312, 0.625, 1.25, 2.5, 5.0, and 10 ng/mL) were used to plot a standard curve. A calibration curve was added to each plate used.

### Body composition evaluation by DXA analysis

DXA was performed by one single experienced technician using a DXA scan (Hologic Inc., Bedford, MA, USA, QDR 4500 W). The coefficient of variation for fat mass (FM) was < 1.5%. Body composition was measured in the whole body and, with the use of specific anatomic landmarks determined by a standard software (Hologic Inc., S/N 47168 VER. 11.2), in the trunk, which included neck, chest, abdominal, and pelvic areas. The upper perimeter was the inferior edge of the chin and the lower borders intersect the middle of the femoral necks without touching the brim of the pelvis. Scans were performed according to the manufacturer's instructions.

### Determination of liver adiposity

The determination of liver fat content was based on liver-kidney contrast measured with ultrasonography by one single trained radiologist with extensive experience in abdominal ultrasound examinations. The analysis was carried out using a Esaote Medica apparatus equipped with a convex 3.5 MHz probe (Esaote MyLab40, Esaote Europe B.V., The Netherlands). The severity of liver adiposity was based according to the brightness of the liver estimated as a numerical value: 0 = absent; 1 = mild lipid accumulation; and 2 = moderate/severe lipid accumulation.

### Echocardiographic epicardial fat thickness measurements

Epicardial Fat Thickness (EFT) was measured through a validated echocardiographic procedure (21). Participants underwent high-resolution M-B-mode transthoracic echocardiography using a 2.5-MHz probe, and spectral Doppler exam of the common carotid artery using a 7.5- MHz probe (Esaote MyLab40, Esaote Europe B.V., The Netherlands). The EFT was identified as the echo-free space between the outer wall of the myocardium and the visceral layer of the pericardium, and its thickness was measured perpendicularly on the free wall of the right ventricle (RV) at end-systole in three cardiac cycles. The average value of three cardiac cycles from each echocardiographic view was considered. All echocardiograms were recorded by the same experienced operator who was blinded to the other study data.

### Statistical analysis

Variables were expressed as mean ± SD. Differences between groups were analyzed using Student's *T*-test. A matrix correlation among variables was calculated. Each variable, in relation to SIRT1, was tested by the use of regression analyses, taking into account sex and age for their potential confounding effect. Violations of normality of the regression models were tested through the Shapiro-Wilk test. In the stepwise regression analysis, we included significant (*p* < 0.05) predictors from linear regression along with variables deemed important, a priori, on clinical grounds. To avoid colinearity, the correlation between variables was assessed and the more clinically relevant variable of a pair of highly correlated variables was included. To arrive to a parsimonious model, covariates were selected with a stepwise regression procedure using backward elimination. The parameters selected were age, sex, waist circumference, EFT, liver steatosis, HDL-cholesterol, ESR, and basal metabolic rate. All *p*-values presented were two-tailed, and values < 0.05 were considered statistically significant. Data were analyzed with the use of STATISTICA software, version 6.1 (Stat Soft, Inc., Tulsa, Oklahoma).

## Results

The characteristics of the study population, stratified according to the patients BMI, are summarized in Table 1. Statistical significances presented for participant characteristics are all obtained from unadjusted analysis. The mean BMI was 16.22 ± 2.44 kg/m^2^, 23.39 ± 1.24 kg/m^2^, and 40.95 ± 6.83 kg/m^2^ in anorexic patients, normal weight and obesity group, respectively. WC was constantly ≥80 cm in females and ≥94 cm in males affected by obesity. The differences in weight, BMI, WC, EFT, total-FM%, trunk-FM% were statistically significant (*p* < 0.001) across the groups. BMR was significantly higher in obese patients compared to underweight (*p* < 0.0001) and normal-weight (*p* < 0.0001) patients and between underweight and normal-weight patients as well (*p* < 0.05).

**Table 1:** Demographic, anthropometric and clinical characteristics of the patients.

**Variables**	**Underweight (*n* = 21)**	**Normal weight (*n* = 26)**	**Obese subjects (*n* = 75)**
Age (years)	32.42 ± 14.62	42.53 ± 10.97	40.88 ± 12.59
Sex (male/female)	3/18	7/19	19/56
SIRT1 (ng/ml)	3.27 ± 2.98	2.27 ± 1.13	1.36 ± 1.31
Weight (kg)	43.79 ± 10.47	65.44 ± 6.71	114.98 ± 22.56
BMI (kg/m^2^)	16.22 ± 2.44	23.39 ± 1.24	40.95 ± 6.83
WC (cm)	66.76 ± 8.13	76.50 ± 8.61	125.86 ± 15.34
Fat Mass (%)	16.83 ± 6.21	25.90 ± 4.30	40.08 ± 5.34
Truncal Fat Mass (%)	12.04 ± 5.03	19.78 ± 3.06	38.39 ± 5.20
EFT (mm)	4.01 ± 0.62	6.86 ± 0.55	8.64 ± 0.86
Liver steatosis (degrees)^*^	Mild	Absent	Moderate/Severe
FPG (mg/dl)	74.23 ± 8.84	96.84 ± 13.73	102.12 ± 20.82
Insulin (μIU/ml)	5.98 ± 3.90	8.97 ± 3.34	17.25 ± 13.65
HDL-C (mg/dl)	72.71 ± 15.81	50.38 ± 17.23	48.67 ± 12.90
LDL-C (mg/dl)	90.80 ± 48.60	103.73 ± 25.96	122.65 ± 28.62
Triglycerides (mg/dl)	99.52 ± 65.85	111.61 ± 44.24	139.62 ± 63.23
ESR (mm/h)	12.70 ± 11.03	22.80 ± 7.84	31.58 ± 18.14
CRP (μg/L)	712.5 ± 788.7	5065 ± 2358.6	6662.6 ± 4221.9
Fibrinogen (g/L)	2.89 ± 0.57	3.50 ± 0.70	3.84 ± 0.77
BMR (kcal/day)	1245.1 ± 149.3	1389.0 ± 303.8	1964.5 ± 349.7

*SIRT1, sirtuin1; BMI, body mass index; WC, waist circumference; EFT, epicardial fat thickness; FPG, fasting plasma glucose; HDL-C, high-density lipoprotein-cholesterol; LDL-C, low-density lipoprotein-cholesterol; ESR, erythrocyte sedimentation rate; CRP, C-reactive protein; BMR, basal metabolic rate. Values are expressed as means ± SD*.

* *The severity of liver adiposity was based according to the brightness of the liver estimated as a numerical value: 0 = absent; 1 = mild lipid accumulation; and 2 = moderate/severe lipid accumulation. For each variable, missing values were < 2%. Information on missing values is therefore not provided in the table*.

### Circulating SIRT1 levels

Underweight patients showed the highest values of SIRT1 followed by normal-weight and obese individuals. The differences in SIRT1 levels were statistically significant between obese subjects and both normal-weight (*p* = 0.002) and underweight patients (*p* < 0.0001).

### Fat amount and distribution

The characteristics of the adiposity of the patients are summarized in Table 1. EFT, total FM % and truncal FM % were significantly reduced in underweight patients compared to both normal-weight subjects and patients affected by obesity (*p* < 0.001).

Both underweight and obese patients had an abnormally high accumulation of liver fat evaluated by ultrasonography compared to normal-weight. However, the degree of liver steatosis was significantly lower in underweight patients (mild degree) compared to obese patients (moderate/severe degree) (*p* < 0.0001).

### Metabolic and inflammatory parameters

There were important metabolic differences between the categories of patients (Table 1). FPG was lower in underweight patients compared to normal-weight and obese patients (*p* < 0.0001). As expected, the highest basal insulin was found in the obesity group. The differences in insulin levels between underweight and normal-weight subjects (*p* = 0.013), and between normal-weight and obese patients (*p* = 0.002) were statistically significant.

LDL-cholesterol levels were comparable in underweight and normal-weight patients, while obese individuals showed higher values of both total and LDL-C (*p* < 0.05). Indeed, HDL-C was higher in anorexic patients compared to normal-weight (*p* < 0.0001) and obese (*p* < 0.0001), while there were not differences between normal-weight and obese patients (*p* = 0.534).

Analogously, the triglycerides concentrations did not differ between underweight and normal-weight subjects (*p* = 0.45), but were significantly higher in patient affected by obesity compared to normal-weight individuals (*p* = 0.03). All the markers of inflammation followed a clear pattern with a statistical significant increase from underweight, to normal-weight, to obese patients.

### Regression analysis

Table 2 shows the regression analysis results for each predictor variable in relation to SIRT1 adjusted for age and sex. SIRT1 was inversely associated with EFT, total FM%, liver steatosis, body weight, BMI, and WC. Concerning the metabolic variables, SIRT1 was negatively associated with LDL-cholesterol, insulin, and BMR. Finally, SIRT1 was inversely correlated with ESR.

**Table 2:** Age- and sex-adjusted linear regression analysis of SIRT1.

**Variables**	**β Coeff**.	**SE**	***p***
Weight (kg)	−0.02	0.00	< 0.001
BMI (kg/m^2^)	−0.055	0.01	< 0.001
WC (cm)	−0.026	0.01	< 0.001
Fat Mass (%)	−0.060	0.02	< 0.001
Truncal Fat Mass (%)	0.04	0.04	0.34
EFT (mm)	−0.396	0.07	< 0.001
Liver steatosis (degrees)	−0.585	0.23	0.01
FPG (mg/dl)	−0.010	0.01	0.26
Insulin (μIU/ml)	−0.028	0.01	0.03
HDL-C (mg/dl)	0.019	0.01	0.07
LDL-C (mg/dl)	−0.011	0.00	0.03
Triglycerides (mg/dl)	0.00	0.00	0.36
ESR (mm/h)	−0.024	0.01	0.02
CRP (μg/L)	0.000	0.00	0.07
Fibrinogen (g/L)	−0.38	0.20	0.06
BMR (kcal/day)	−0.002	0.00	< 0.001

*BMI, body mass index; WC, waist circumference; EFT, epicardial fat thickness; FPG, fasting plasma glucose; HDL-C, high-density lipoprotein-cholesterol; LDL-C, low-density lipoprotein-cholesterol; ESR, erythrocyte sedimentation rate; CRP, C-reactive protein; BMR, basal metabolic rate*.

There was no significant association between SIRT1 and triglycerides, HDL-cholesterol, fasting glycaemia, trunk FM%, fibrinogen and CRP.

Given that metabolic and inflammatory markers are influenced by degree of adiposity, we ran an additional set of analyses that included adjustment for WC, beyond age and sex, to assess whether the associations observed for SIRT1 were independent from adiposity. We found that the association between SIRT1 and either inflammatory (ESR, CRP, fibrinogen) or metabolic (FPG, insulin, HDL-cholesterol, LDL-cholesterol, triglycerides) parameters was abolished once adjusted for WC, suggesting that the major drive for the variation of circulating SIRT1 levels is the adiposity *per se* (data not shown). WC was adjusted for because WC is a reliable representative of adiposity and SIRT1 expression parallels visceral fat.

### Backward stepwise regression analysis

Multivariate stepwise regression analysis was used to identify factors that influence circulating SIRT1 across AN, obese and normal-weight groups. We considered only a sub-set of the variables initially tested in linear regressions for the backward stepwise analysis (age, sex, WC, EFT, liver steatosis, HDL-cholesterol, ESR and BMR), depending on both preliminary statistics and clinical appraisal.

The results from the analysis provide the set of independent variables that best explain the variance in plasma SIRT1 levels in the current sample, although the results are limited by the small sample size. In the study population, age and EFT were the sole determinants of circulating SIRT1 with a β-coefficient of −0.026 (*p* = 0.025) and −0.516 (*p* = < 0.001), respectively, and a *R*^2^ value of 0.3698 (Table 3).

**Table 3:** Stepwise multiple regression analysis results to identify predictor variables associated with circulating SIRT1 (ng/ml).

**Variables**	**β Coeff**.	**SE**	***p***
Age (yr)	−0.026	0.011	0.025
EFT (mm)	−0.516	0.083	< 0.001

*EFT, epicardial fat thickness. Variables included in the starting model for circulating SIRT1 were: age, sex, waist circumference, EFT, liver steatosis, high-density lipoprotein cholesterol, erythrocyte sedimentation rate and basal metabolic rate. We left in the model all variables that met the 0.15 significance level for entry into the stepwise model. R-squared value = 0.3698*.

## Discussion

**Figure 1:**
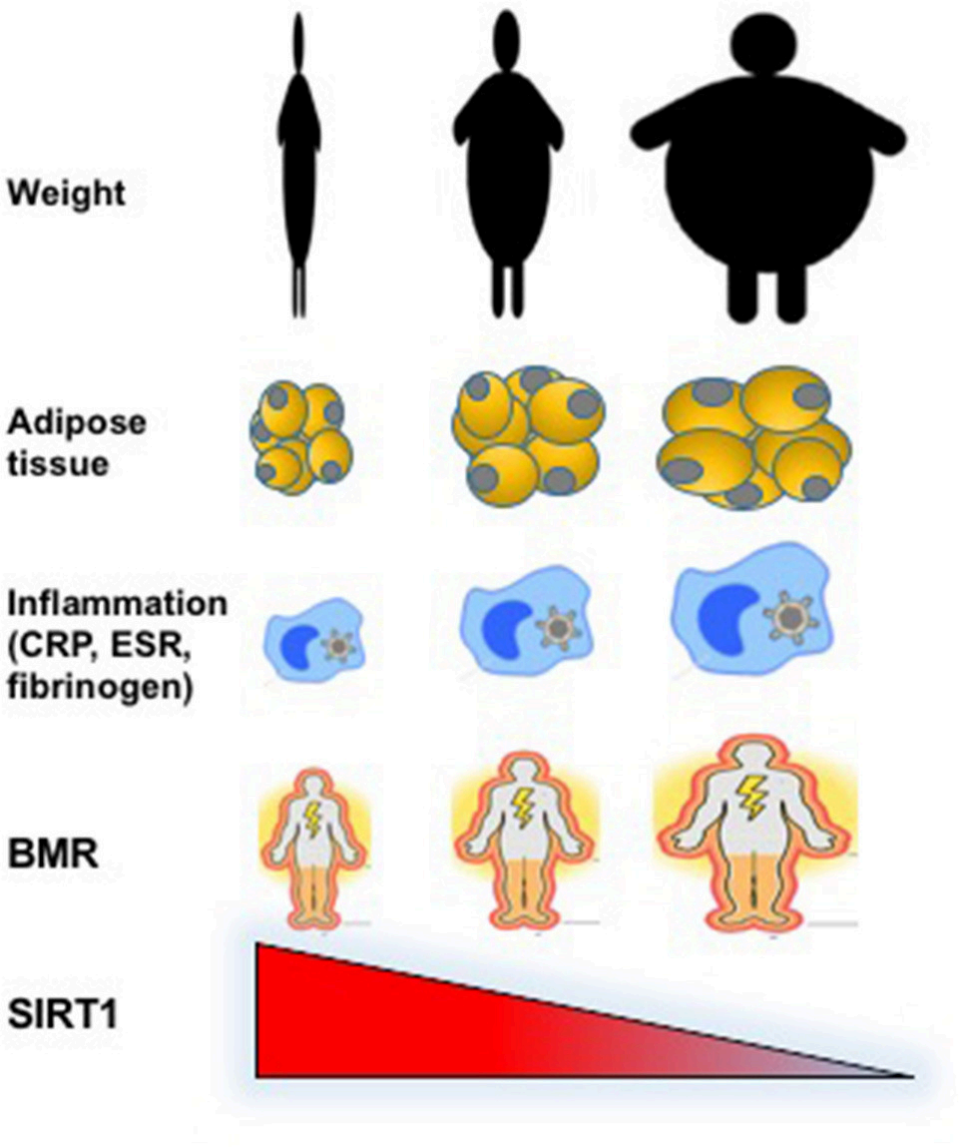
Graphical representation of the inverse relationship between circulating levels of SIRT1 and weight, fat abundance and distribution, inflammatory, and metabolic parameters in underweight, normal weight, and obese individuals.

In this study, we compared the circulating levels of SIRT1 in condition of deficiency, normal content or excess body fat in underweight, normal-weight, and obese patients. We found a significant negative correlation between plasma SIRT1 and adipose tissue, with the highest levels observed in participants with extremely reduced fat content (Figure 1). This observation is novel and opens new questions dealing with the regulation of SIRT1 production and its function in relation to adipose tissue.

Several studies have provided insights into the mechanisms underlying endocrine, metabolic, and adaptive changes in states of chronic starvation (22).

SIRT1 concentrations were negatively correlated with insulinemia and LDL-cholesterol. This is likely explained by the compelling evidence that SIRT1 overexpression offers substantial benefits on serum cholesterol and insulin levels and increased resistance to high-fat diet induced glucose intolerance and insulin resistance (23–25). No association was found between SIRT1 levels and triglycerides, although contradictory results have been obtained with resveratrol, a potent SIRT1 activator, that was found either to reduce (26) or to have no effects (27) on plasma triglycerides.

SIRT1 is an essential negative inflammatory regulator in high-fat diet or alcohol induced fatty liver diseases, mainly through deacetylating NF-κB and down-modulating NF-κB transcriptional activity, thereby reducing macrophage infiltration and pro-inflammatory cytokines production in the liver as well as in the adipose tissue (28). Indeed, in line with other studies (29, 30), we found that underweight patients were less inflamed compared to normal-weight and obese patients and ESR was inversely associated to the pattern of SIRT1 and proportional to fat mass. This coincides with the assumption that obese patients, generally, show a pro-inflammatory phenotype and express less SIRT1 than lean subjects.

It is worth to be mentioned that additional adjusted regression models for WC, a reliable predictor of visceral adiposity, abolished the association between SIRT1 and inflammatory and metabolic parameters, indicating that fat content is the most relevant determinant of SIRT1 circulating levels in this study.

In the stepwise regression analysis, epicardial fat, out of all the markers of adiposity included in the study whose expression is linearly associated with SIRT1, is the variable most strongly associated with SIRT1. The strict association between EFT and blood SIRT1 was not unexpected, being already seen previously (8). But, then again, human studies have shown that SIRT1 is expressed in visceral adipose tissue and reduced by obesity (31) and echocardiographic measurement of epicardial fat can provide a more specific and sensitive measurement of intraabdominal visceral fat (32). Therefore, a possible explanation for the preferred association between EFT and SIRT1 might depend on the robust representativeness of epicardial fat as visceral fat as opposed to other measures of adiposity used and SIRT1. In line with previous studies (8), partly based on the same study sample, the inverse relationship between SIRT1 and EFT adds a new potential mechanism to the evidence supporting the role of epicardial adipose tissue in the development of atherosclerosis and its complications, inflammation, and metabolic syndrome in obese patients.

Furthermore, it is relevant the negative association seen between age and circulating SIRT1 levels. This observation confirms what previously reported in a comprehensive study aimed at identifying the pattern of serum SIRT1 activity according to age (33).

Individuals with AN have lower resting energy expenditure than normal-weight controls (34) and CR is a powerful stimulus for SIRT1 activation (18), likely an adaptive mechanism to preserve energy for vital functions. Accordingly, in SIRT1 gain-of-function transgenic mice, SIRT1 behaves as a “*thrifty gene*” that protects against metabolic diseases by instructing the organism to limit energy consumption and expenditure (23). Although our data are purely associative in nature, they seem to confirm the hypothesis that SIRT1 levels have the tendency to match with energy saving since the higher the SIRT1 values the lower the BMR values. Further studies to reveal the relationship between SIRT1 and BMR are warranted.

Although dysregulations of peripheral adipokines, gut-secreted peptides and central neurotransmitters involved in appetite modulation have been detected in patients with AN (35), the significance of these derangements for the development, course and prognosis of eating disorders is still not clear. Actually, there are no conclusive data as to whether alterations of feeding regulatory substances precede the appearance of an eating disorder or are the consequence of the nutritional aberrations occurring in the disorder. It has been suggested, although not definitively proved, that those alterations, even when secondary to malnutrition and/or to aberrant eating behaviors, might contribute to the genesis and the maintenance of some symptomatic aspects of AN, thus affecting the course and the prognosis of the disease. Whether the high levels of circulating SIRT1 in AN individuals is a consequence of the feeding behavior of these patients and whether they may modulate eating-related or non-eating-related psychopathological aspects of AN deserve to be deeply investigated. Interestingly, hypothalamic SIRT1 stimulates food intake and weight gain (36), raising the hypothesis that forms of AN might associate with SIRT resistance. These considerations may be an interesting starting point to study whether SIRT resistance might play a role in the pathogenesis of AN.

SIRT1 is found in a wide range of tissues and organs, highly expressed in liver and adipose tissue and regulated by nutritional status. In general CR stimulates SIRT expression (17, 18, 37) while high calorie diet reduces it. Thus, SIRT1 tissue expression and activity is influenced by the availability of energy suggesting that SIRT1 could have a role in the regulation of normal energy balance. Accordingly, plasma SIRT1 levels and fat mass are inversely regulated, with SIRT1 concentrations being increased in a catabolic condition and decreased in conditions of extreme BMIs.

Remarkably, CR dependent changes occur in a highly tissue-specific manner, as demonstrated by comparing circadian gene expression in the liver vs. epidermal and skeletal muscle stem cells (38). *De novo* oscillating genes under CR show an enrichment in SIRT1 targets in the liver due to enhanced SIRT1 activity (39). Therefore, we hypothesize that the increased circulating SIRT1 levels recorded in severely underweight patients may reflect the reorganization of metabolic pathway linked to SIRT1 in the liver of calorie restricted anorexic individuals.

The measurement of the circulating SIRT1 in severely underweight patients may provide new pathogenetic hypothesis for some of the features of AN.

Limitations of our study are the relatively small number of study subgroups and the use of calculated BMR values. Moreover, males and females were not analyzed separately because of the scarcity of males in our sample. We recognize that our results and conclusions are based on observational data and that the associations between SIRT1 levels and the variables measured do not establish causative roles. The strength of our study is the separation and comparison of different weight subgroups.

In conclusion, circulating SIRT1 inversely parallels the entire spectrum of fat phenotype, basal metabolic rate, inflammatory status, and eating behavior from anorexia to obesity through normal weight.

## Ethics statement

This study was carried out in accordance with the recommendations of the guidelines, Ethical Committee of the University of Rome La Sapienza with written informed consent from all subjects. All subjects gave written informed consent in accordance with the Declaration of Helsinki. The protocol was approved by the ethical committee of the Sapienza University of Rome, Policlinico Umberto I.

## Author contributions

SM and LG conceived the project, developed the overall research plan, and wrote the manuscript. PM, AP, and AS selected the patients and collected the patients' data. MdG made the ultrasound determination of liver adiposity. GB made the echocardiographic epicardial fat thickness measurements. SB and SC made the circulating SIRT1 assay. SM, EP, and AR made the statistical analysis. SM, LG, AG, CL, LD, and AL interpreted the data and critically revised the manuscript for important intellectual content. All authors read and approved the final manuscript.

### Conflict of interest statement

The authors declare that the research was conducted in the absence of any commercial or financial relationships that could be construed as a potential conflict of interest.
